# The Essence and Transcendence of Scientific Publishing

**DOI:** 10.3389/frma.2022.822453

**Published:** 2022-02-16

**Authors:** José L. Medina-Franco, Edgar López-López

**Affiliations:** ^1^DIFACQUIM Research Group, Department of Pharmacy, School of Chemistry, Universidad Nacional Autónoma de México, Mexico City, Mexico; ^2^Department of Chemistry and Graduate Program in Pharmacology, Center for Research and Advanced Studies of the National Polytechnic Institute, Mexico City, Mexico

**Keywords:** dissemination, ethics, open science, publishing, scientific writing

## Introduction

Disseminating the results of scientific research in various forms (typically, peer-reviewed papers, conferences, and so on) nurtures and shapes the advancement of science. Scientific publishing is highly attached to the four well-known Mertonian norms and values that comprise the character or ethos of modern science, namely: communism, universalism, disinterestedness, and organized skepticism (Merton, 1973). This is particularly true for publications that follow a rigorous peer-review and editorial process. Alongside dissemination of science that arguably is the primary reason for scientific publishing, it has other scientific, academic, and professional benefits on the large. However, the pressure of publishing as a documented proof of productivity in academic and other professional settings has led to the “publish or perish' aphorism (Neill, 2008; Publish or perish, 2010). In many cases, such paradigms obscure or alter the actual reasons and motivations for publishing, making it a “survival mechanism.”

This manuscript aims to share the authors' opinions and revisit the *right* and fundamental reasons for scientific publishing. This Opinion is mainly directed to the students and young researchers that sometimes struggle at the beginning to organize, plan, and develop a manuscript. The younger generations (and other more advanced or senior researchers) should consider scientific publishing as more than a survival mechanism to not perish, because such a focused motivation is counterproductive and burdens these initial steps. Here, we highlight several other valid and collateral reasons for publishing beyond academic survival. Other important aspects of scientific publishing are not addressed in detail here, such as peer-review, the cost associated with open access, metrics to evaluate and rank the journals' quality, and ethics in publishing. Instead, they are mentioned and discussed in the context of the primary goals and collateral benefits of publishing.

## The Right Reasons for Scientific Publishing

### Primary Goals

Communication and publishing have been central to advancing science. Indeed, the publishing landscape has changed dramatically in response to technological progress. For instance, over the last few decades, we have witnessed a rapid evolution from printed journals and mailing hard copies to libraries, research offices, etc., to instant online access to the scientific literature digitally, as long as the internet is available. Nowadays, the number of journals solely accessible online is rapidly increasing, as is the number of open access journals (Strawn, 2021). Regardless of the publishing mechanism, the primary goal is to disseminate new results, discuss that information, and ultimately generate knowledge. Also, publishing new results help to revisit (correct or refine) models or previous hypothesis and theories. In some instances, the concept might not have an immediate impact (sometimes because the community is distracted pursuing fashion-driven research or are working on research programs that are receiving funding), but application of the concept can have a major impact (Ke et al., 2015). In any case, proper documentation, support, and description of the methods, theories, etc., are imperative.

Table 1 summarizes a shortlist of primary goals and collateral benefits of scientific publishing. Scientific journals have different manuscript types to classify better or accommodate the variety of contributions that a paper can have. The collateral benefits are positive, and it is highly desirable to achieve them in parallel to the primary goals. However, issues arise when the collateral or secondary goals become the primary. In other words, when the mindset of the student or researcher is “publish” to get something (the Ph.D. degree, a postdoc position, a job or permanent position, funding) and it might become a pure survival goal. Moreover, such survival motivations are often not enjoyable and can introduce opportunities for scientific misconduct and other aspects of the dark side of scientific publishing, as commented in the next section.

**Table 1:** Examples of primary and collateral benefits of scientific publishing.

**Goal, type of contribution**	**Type of manuscript**
**Primary goals**	
• Disseminate new results. • Propose novel theories. • Support with evidence (data), generalization of currently accepted theories. • Refine, adjust, and correct theories, knowledge.	• Research papers. • Full or original papers. • Letters. • Rapid or short communications.
• Disseminate scientific and technological advances. • Develop software, webservers. • Share (curated and high-quality) data.	• Application notes. • Software development/ tools. • Databases.
• Collect, and discuss advances on a specific topic.	• Reviews. • Mini or short reviews.
• Tackle problems from different angles and points; comments, suggestions. • Share opinions on a topic based on own and other research groups' views.	• Commentaries. • Opinion manuscripts. • Viewpoints. • Perspectives.
• Present and discuss a new or established protocol to make it clear and reproducible. • Teach concepts and methods.	• Methods. • Tutorials. • Educational type papers.
**Benefit**	**Type of goal**
**Examples of collateral benefits of scientific publishing**	
• Support application to be admitted in an academic program e.g., Masters or Ph.D. program in an institution in a different country that might not be familiarized with the grading scales of the own university or school. • Get a Ph.D. degree: most institutions require at least one paper in a quality peer-reviewed journal. • Help to obtain scholarships.	Academic
• Contribute to securing a position, e.g., postdoctoral researcher. • Support grant applications. • Help to get tenure. • Obtain a promotion (and better salary). • Obtain favorable performance evaluations. • Help support applications for awards and get funding to continue the research.	Career advancement and funding
• Continued improvement of the dissemination of results and contributions as outlined above in this table. • Help improve research planning and organization of results. • Motivate learning and self-improvement. • Develop the ability to take and respond to objective and constructive criticisms. • Improve scientific writing skills. • Improve skills to work in collaboration with other members of the research group or external collaborators. The latter point is particularly relevant in multidisciplinary research groups where effective communication is essential^a^.	Professional advancement

a *The ability to work effectively in a research environment is not always easy and sometimes is the driving force of the project's failure (Medina-Franco, 2021)*.

## The Gray-to-Dark Side of Scientific Publishing

The publish or perish notion has negative “gray” or “dark” sides. Harmful and controversial publishing practices frequently driven by the need to survive in academia can be intentional or non-intentional (i.e., “honest mistakes”). Some others are inherent to the human factor. Examples of the gray-to-dark sides of scientific publishing are briefly commented hereunder.

### Scientific Misconduct and Bad Practices in Publishing

Although it is not the primary goal of this manuscript to discuss the bad practices in publishing, which have been thoroughly documents and debated by the Committee on Publication Ethics—COPE (Wager, 2012), these practices are a critical component of the topic area. Common bad practices in publishing include but are not limited to: plagiarism and self-plagiarisms; data fabrication; figure manipulation; predatory publishing; and slicing the same study into different smaller parts to increase the number of publications (the so-called “salami” publishing). Other challenging issues are associated with authorship and include addition of high profile authors as “honorary” co-authors tying to publish in a highly ranked journal; authors' unjustified groups of co-authors to increase the number of publications of research groups; including authors that did not contribute to the work for conflicts of interest; and other unethical authorship practices. Another serious problem is the potential for “paper mills,” where manuscripts are manufactured with fake or altered data and figures, and plagiarism, among other ethically challenged practices (Wager, 2012).

### Ego and “Academic Greediness”

Improving research ensuring quality of each research study and resulting manuscript is a desirable goal for every student and researcher. Likewise, the opportunity to contribute to the prestige associated with a research institution or country of origin, is also, we believe, a positive and good motivation for publishing. However, self-prestige (or ego) is very human characteristic that can darken the motives of scientific publishing. The so-called “academic greediness” (e.g., increasing the number of publications, citations, etc.) purely by an ego-driven desire to attain high numbers of papers, can obscure the goals and the essence of publishing. Academic greediness can be considered the opposite of Merton's disinterestedness norm (*vide supra*) (Merton, 1973). Such typical human factors are rooted in a desire to be noticed or accepted but the community just for the sake of prestige, and can play a role in the practices of some publishers to increase the costs of publishing in that journal because researchers may feel it is worth any price (even from their own pocket) to have the reputation of publishing in X prestigious journal.” Sadly, that “price” might motivate some scientists to other ethical misconducts such as data fabrication and manipulation. Even if ethical misconduct is not performed, just publishing in a prestigious journal only for the sake of personal gain, is against the ethos of science (Merton, 1973).

## Training of Young Researchers and Students

As part of the integral academic and professional development the students in their research groups, heads of research groups, and head of academic programs, in general, should promote an ethical scientific culture. i.e., not only trying to encourage students to participate in research projects that engage the curiosity, interest, and passion, but also doing it ethically for the right motivations discussed in the previous sections. These practices could help prevent scientific misconduct in research. For example, the unfortunate but common practice of copying and cheating in exams to get a benefit (e.g., pass an exam), if not corrected, can manifest, for example, as plagiarism to also get a benefit (i.e., a paper published). Additionally, investments in the development of early career researchers, including their ethical and professional development, has proven to positively impact the quality and quantity of scientific publications (McGrail et al., 2006). Finally, it is essential to focus on the main drivers of scientific publishing, addressing questions such as what are the main findings and contributions of the research work? or what is the significance of the results? Instead of focusing on questions such as in what journal were the results published? Or what is the journal ranking? The later questions are relevant but should not be the primary concern of a scientific manuscript.

## Conclusions

Why publishing? Must we publish? What happens to my scientific career if I do not publish frequently enough? These are examples of frequent and valid questions that everyone in the scientific career asks themselves at some point. Such questions *seem* to be partially answered by the scaring aphorism “publish or perish.” Such interrogatives emerge again when one faces all the hurdles associated with planning and producing a manuscript, followed by the sometimes subjective and imperfect peer-review process. By focusing on the right reasons for publishing, one can find excellent and valid motivations to go through this challenging process. Even so, this process is rewarding and fascinating if it is done for the right and ethical reasons.

In this manuscript, we share the authors' opinions on what we refer to as primary goals and collateral benefits of scientific publishing. The primary goals are related to the dissemination and progress of science fulfilling the Mertonian norms. The collateral benefits include professional development and personal growth and competitive advantages for secure scholarships and research funding. The dark side of scientific publishing is dominated by scientific misconduct and bad publishing practices, which can be driven when the collateral benefits become the primary goal of the scientist to publish. To be clear, we are not against trying to publish in top-ranked, well-known, and prestigious journals if the motive for doing so serves to disseminate one's research and advance the discussion in a field, thereby making other outstanding contributions (aka, right reasons).

As academics, we should encourage students and young generations to adjust the mindset and consider publications beyond a mechanism or survival. Instead, use the true value of publishing as an opportunity to help the advancement of research and science.

## Author Contributions

Both authors contributed to the writing of the manuscript.

## Conflict of Interest

The authors declare that the research was conducted in the absence of any commercial or financial relationships that could be construed as a potential conflict of interest.

## Publisher's Note

All claims expressed in this article are solely those of the authors and do not necessarily represent those of their affiliated organizations, or those of the publisher, the editors and the reviewers. Any product that may be evaluated in this article, or claim that may be made by its manufacturer, is not guaranteed or endorsed by the publisher.
